# Successful Strategy for the Conservative Management of Acquired Tracheoesophageal Fistula Due to Lithium Button Battery Ingestion

**DOI:** 10.1055/s-0040-1705157

**Published:** 2020-04-14

**Authors:** Soichi Shibuya, Takahiro Azuma, Geoffrey J. Lane, Manabu Okawada, Atsuyuki Yamataka

**Affiliations:** 1 Department of Pediatric General and Urogenital Surgery, Juntendo University, Tokyo, Japan

**Keywords:** foreign body ingestions, tracheoesophageal fistula, conservative treatment, lithium button battery, mediastinitis

## Abstract

A 16-month-old boy was referred to our hospital for the management of suspected lithium button battery (LBB) ingestion. He had been previously well, but became febrile with a persistent cough resistant to oral antibiotics and dysphagia for 5 days. Radiography identified an LBB lodged in the upper esophagus. The LBB was retrieved under direct visualization with rigid laryngoscopy. He was sedated for 5 days and enteral feeding was commenced through a nasojejunal tube on the next day after procedure. On day 8 after retrieval, endoscopy and fluoroscopy identified a tracheoesophageal fistula (TEF), 6 mm in diameter. Conservative management was conducted with periodic follow-up endoscopies, which showed signs of healing in the esophagus. Following continuous antibiotics and proactive nutritional support, the TEF was found to have closed spontaneously by day 28 after the LBB removal. We present our experience of the successful nonsurgical management of acquired TEF secondary to LBB ingestion and aim to establish a protocol for managing it conservatively by reviewing the relevant literature.

## Introduction

Small children have a strong interest to small objects and easily get tempted to swallow them, making emergency rooms frequently encounter accidental foreign body ingestions. Thankfully, most foreign body ingestions are harmless and can be observed safely, but some circumstances demand immediate treatment and surgical intervention. Lithium button battery (LBB) ingestion is one of the situations that requires prompt surgical consultation, especially if the ingested battery is greater than 20 mm in size and 3 V in power, because of its high morbidity and even potential mortality. 1 2 3 4 5 Among possible complications of LBB ingestion, tracheoesophageal fistula (TEF) is particularly disastrous and sometimes causes catastrophic consequence. Traditionally, acquired TEF is managed with surgical intervention, but conservative management is the first-choice treatment at our institution. We present a case of TEF following LBB ingestion that was treated successfully by nonsurgical therapy, discuss the strategy, and suggest indications for conservative management.

## Case Report

**Fig. 1:**
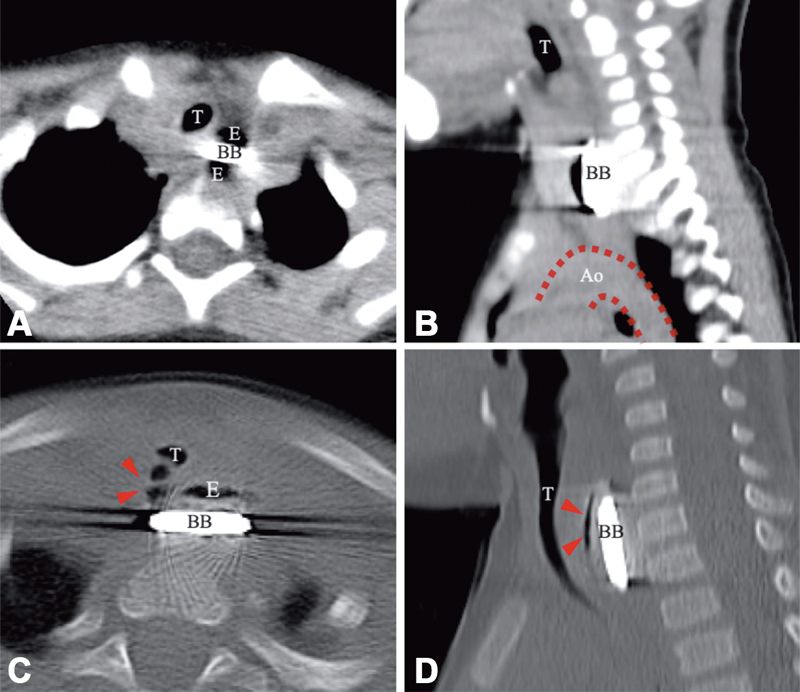
Computed tomography showing focal air in the mediastinum suggesting esophageal perforation. ( **A** ) Axial image—The cathode side faced forward and the BB was tilted slightly clockwise. ( **B** ) Sagittal image—There was only 1cm between the BB and the Ao. ( **C** ) Mediastinal window, axial image—Arrowheads indicate focal air in the mediastinum. ( **D** ) Mediastinal window, sagittal image—Arrowheads indicate focal air in the mediastinum. Ao, aorta; BB, button battery; E, esophagus; T, trachea.

**Fig. 2:**
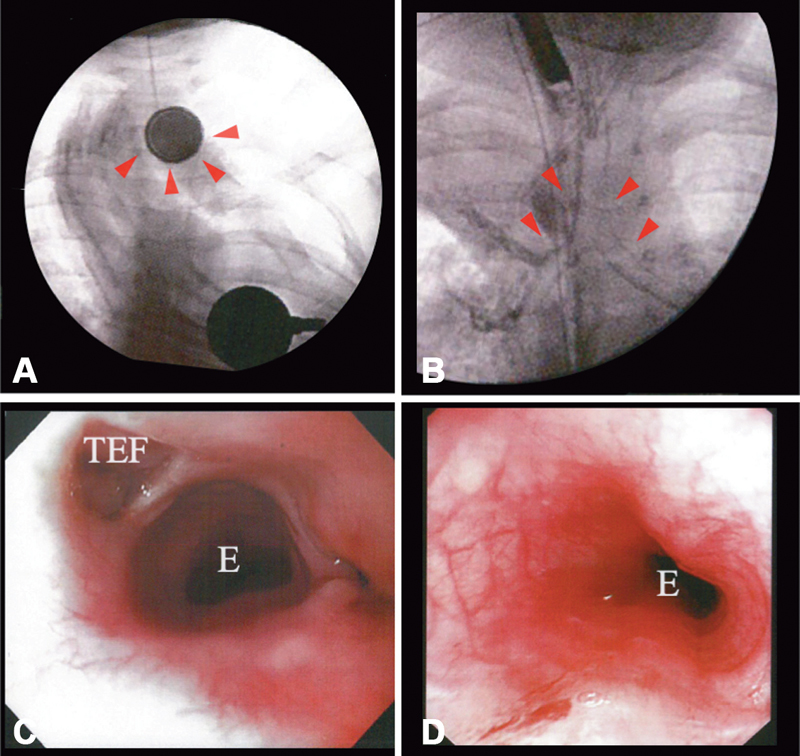
( **A** ) Fluoroscopy at the time of referral arrowheads indicated a lithium BB impacted the upper esophagus. ( **B** ) Contrast fluoroscopy on 8 days after retrieval. Arrowheads indicate contrast agent flowing into the trachea. ( **C** ) Flexible endoscopy on 15 days after retrieval. Flexible esophagostomy revealed TEF formation, 6 mm in diameter. ( **D** ) Endoscopy on 28 days after retrieval. Spontaneous closure of TEF was confirmed. E, esophagus; TEF, tracheoesophageal fistula.

A previously healthy 16-month-old boy was referred to our hospital for the management of suspected LBB ingestion. He began to refuse food 5 days before referral and developed persistent cough and high fever 2 days before referral. His parents took him to see a pediatrician who commenced oral antibiotics, but without improvement. He was taken to another hospital and X-ray identified a round foreign body lodged in the cervical esophagus. Computed tomography (CT) showed focal air in the mediastinum suggesting esophageal perforation ( Fig. 1 ). He was immediately referred to us for further treatment. Emergency rigid laryngoscopy was performed under general anesthesia and identified a 20 mm 3V LBB lodged in the upper esophagus ( Fig. 2A ). The LBB was carefully removed with Magill forceps. Flexible esophagoscopy and contrast esophagoscopy performed after the retrieval demonstrated severe erosion on the esophageal wall, but at that point, a fistula was not identified. The patient was kept intubated for 5 days for the purpose of maintaining a secure airway and preventing the injured area from being exposed to excessive saliva.

Two nasal tubes, a 10 Fr nasogastric tube for stomach decompression and a 6.5Fr nasojejunal tube for enteral feeding, were placed under fluoroscopy. Enteral feeding with liquid hyperalimentary formula (Racol, Otsuka Pharmaceutical Factory, Tokyo, Japan) supplemented with l -arginine was initiated at 60 kcal/kg/day on the day after retrieval and gradually increased to 100 kcal/kg/day. Ampicillin/sulbactam (200 mg/kg/day) was administered and converted to doripenem (60 mg/kg/day) because of an acute febrile episode occurred 5 days after retrieval.

Follow-up esophagoscopy and contrast study on day 8 after retrieval demonstrated an acquired TEF ∼6 mm in diameter at the site where LBB had been impacted ( Fig. 2B , C ). Antibiotic therapy and proactive nutritional support were continued along with periodic follow-up esophagoscopy. The second follow-up endoscopy was performed on day 16 after retrieval and demonstrated the healing process of the esophageal wall, encouraging us to continue the conservative management. After 28 days of careful conservative management, spontaneous closure of the TEF was confirmed by esophagoscopy and esophagography ( Fig. 2D ). He started eating orally on the next day and was discharged on day 40 after retrieval. At 2 years follow-up, he is asymptomatic and growing well without any dysphagia or recurrence.

## Discussion

Recently, button batteries (BB) have become ubiquitous in small electrical devices, increasing the incidence of accidental ingestion. 6 Of these, LBB is especially hazardous with its high potential in voltage delivery and tendency to lodge in the esophagus. Studies that investigate the speed of LBB damage in porcine esophagi have shown mucosal disruption in less than 15 minutes and serious burns to the muscular layers within 2 hours. 4 Of note is that LBB retain their capacity to cause tissue injury even after they can no longer power an electric device because of residual voltage in the battery. 7 8 Although there is no national database in Japan for ingestion of foreign bodies, several surveys suggested increasing morbidity after BB ingestion, especially LBB. Prompted by this trend, the Japanese Society of Pediatric Surgeons and National Consumer Affairs Center of Japan have called on manufacturers and distributors of batteries to improve packaging and made it mandatory to sell LBB in specific child-proof containers. 9

In some cases, parents do not witness the ingestion, resulting in a delayed presentation with symptoms of esophageal injury, such as dysphagia, vomiting, fever, and cough. 5 Buttazzoni et al described that dyspnea, drooling, and vomiting are more common in toddlers, while older children may also complain of abdominal or chest pain. 10 If a previously healthy child has sudden onset of any of these symptoms, BB ingestion should be suspected and chest X-ray is recommended.

For retrieval, we advocate direct extraction with a rigid laryngoscope under general anesthesia. From experience, this is the most straightforward and safe approach when LBB is lodged in the upper esophagus. If ingestion occurred more than 2 hours ago, LBB would already have started to injure the esophageal epithelium, and any attempt at retrieval using balloon extraction or flexible esophagoscopy is not likely to succeed and could inadvertently injure the esophagus. 11 Rigid laryngoscopy can visualize LBB more clearly and facilitate retrieval under direct view. Careful examination of the damaged esophageal wall after extraction is requisite for considering the management following the procedure.

Even after LBB is successfully removed, the patient is still at risk for developing complications. Inflammation can persist in the esophagus and surrounding tissues, making fistula formation likely to occur even more than 24 hours after retrieval. 12 13 14 15 Leinwand et al reported a tragic fatal case in which aortoesophageal fistula was developed, even though LBB had passed through into the stomach and was not impacted in the esophagus at the diagnosis. 11 Therefore, hospitalization for careful observation is mandatory no matter how well the child may appear, and the esophagus must be investigated thoroughly, unless LBB was removed immediately after witnessed ingestion without any evidence of injury during extraction.

Once TEF is diagnoses, treatment strategy must be decided upon. Surgical repair of TEF requires repair of both the esophagus and the trachea through thoracotomy or sternotomy. If direct closure of the fistula is not possible or fails, more invasive approaches, such as gastric transposition and slide tracheoplasty, are next line. Given that these surgical interventions have a high risk of postoperative complications and carry significant morbidity, the potential benefit of successful conservative management is appreciable. 5 16 17 Nevertheless, it must be emphasized that conservative management also has risk; children may suffer from severe mediastinitis and aspiration pneumonia. Deteriorating patient's condition due to hemorrhage or respiratory impairment is the most decisive factor for emergent surgery. CT is useful for revealing the position and direction of LBB and the extent of inflammation, which might determine surgical indication. As necrotic damage occurs around the edge of a buttery rather than the face, and the most severely at the negative pole, the direction of LBB can be a clue to predict the subsequent injury. 2 7 8 Considering the possibility of sudden catastrophic bleeding due to esophagoaortic fistula, active inflammation in close proximity to the aortic artery indicates the inevitability of urgent surgical intervention. 6 The axial and sagittal images in Fig. 1 show the direction of the LBB in the present case and the positional relationship with the esophagus, the trachea, and the aorta. The negative pole faced anteriorly and the LBB was tilted slightly clockwise. This implied that the point of strongest inflammation was close to the trachea, whereas an ∼1 cm distance was kept between the lowest edge of the LBB and the aorta. Had the BB lodged more inferiorly and the negative pole faced the aorta, we would have chosen surgical intervention rather than conservative management.

We conducted literature search in the PubMed database using the terms “button battery” AND “tracheoesophageal fistula” during the period from 1989 to 2019. Thirty-two English publications were identified and reviewed. References from the papers were also manually searched and, if relevant, included in review process. From the reviewed papers, reports describing an attempt at conservative treatment for acquired TEF after LBB ingestion were selected and tabulated in Table 1 . 11 12 13 18 19 20 Cases with TEF caused by mercury or alkali BBs were excluded, leaving only limited reports related to LBB ingestion. 18 21 22 23 24 The reported cases of TEF due to LBB ingestion demonstrate these TEF to be more complicated and prone to surgical intervention than other type of BBs. There were two cases in which conservative management failed and surgical intervention was eventually performed. 18 19 Although it was difficult to compare successful and unsuccessful conservative management being limited by the small number of reported cases, there was no apparent difference in the age of the patients, the duration of impaction, and size of the fistula. In the unsuccessful cases, diagnosis of TEF was established relatively later after removal of LBB, and their episodes of TEF formation and recurrence were presumably associated with oral feeding. In the case of Alkan et al, the patient was allowed to eat orally before return to the hospital with symptoms of dysphagia, fever, cough, and drooling of saliva 1 week later after discharge. 18 In the other report, Grisel et al took more vigilant approach and restricted oral feeding to pure diet only, but the patient readmitted 2 days after discharge with TEF formation. 19 Notably, a recurrence of symptoms occurred 4 days after they initiated oral intake following ascertainment of spontaneous closure of TEF. Based on lessons learned from these reports, we maintain a stringent nil by mouth policy until we confirmed that the fistula was completely cured.

**Table 1:** Attempted conservative management of acquired TEF after accidental lithium button battery ingestion

Authors published	Age at ingestion Sex	Duration of ingestion	Diagnosis of acquired TEF	TEF size	Management	Duration of healing	Hospital Discharge	Outcome
Senthilkumaran et al 1996	5 mo M	12 d	Soon after removal	Unk	CV nutrition, NJ feeding	6 wk	Unk	Unremarkable
Chiang and Chen 2000	20 mo M	3 d	On presentation	Unk	NJ and NG feeding, antibiotics, steroids	11 wk	4 wk	Unremarkable
Anand et al 2002	41 mo M	10 d	1 d	Unk	NG drainage, NG feeding, antibiotics	28 d	Unk	Unremarkable
Alkan et al 2004	16 mo F	3 d	15 d	Unk	Gastrostomy, CV nutrition. antibiotics	Not closed after 5 wk	Unk	Subsequent surgery
Grisel et al 2008	3 y F	12 h	12 d	9 mm	GJ feeding, PPI	Recurrent after 80 d	Unk	Subsequent surgery
Russell et al 2013	11 mo M	6 h	7 d	7 mm	NG drainage, NG feeding, antibiotics	1 mo	53 d	Unremarkable
The present case 2017	16 mo M	5 d	8 d	6 mm	NG drainage, NJ feeding, antibiotics, PPI	28 d	48 d	Unremarkable

Abbreviations: CV, central venous catheter; NG, nasogastric; NJ, nasojejunal; PPI, proton-pump inhibitor; TEF, tracheoesophageal fistula; Unk, unknown.

**Fig. 3:**
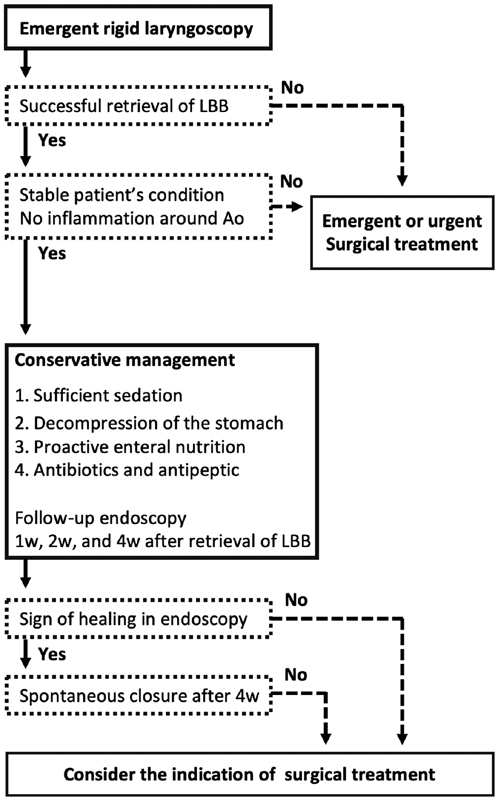
Flowchart for the conservative management of TEF after LBB ingestion. Ao: aorta; LBB, lithium button battery; w, week.

We summarized our treatment strategy for TEF due to LBB ingestion in a flowchart ( Fig. 3 ). Our protocol for the conservative management consists of:

1. Sufficient sedation after retrieval of LBB.
2. Decompression of the esophagus and the stomach.
3. Proactive enteral nutrition (postpyloric).
4. Appropriate antibiotics and antipeptic therapy.

Life-threatening respiratory failure can occur soon after retrieval due to obstructed airway associated with edematous larynx or aspiration pneumonia through TEF. To secure the safe airway in the presence of TEF, we keep patients intubated under deep sedation after retrieval. We hypothesize that this process protects the trachea from inflammation and exposure to continuous air flow too early, while the trachea my still be somewhat sensitive. Nevertheless, the duration of deep sedation we advocate is not based on any trial evidence; rather, it is a decision based on years of experience. To what extent deep sedation contributes to the success of conservative management is unclear; therefore, sedation protocol may be modified at the discretion of management teams. Managing a patient without intubation using mild sedation protocol might be another choice to avoid ventilator-associated complications. In any case, oral intake should be restrained until the state of inflammation in the esophageal wall is evaluated by follow-up endoscopy.

In the presence of TEF, aspirated air flows into the digestive tract and inflates the stomach, resulting in raised intragastric pressure and reflux of gastric acid that can be detrimental to the healing process of the esophagus. 20 Therefore, sufficient decompression of the stomach is key. Some authors recommend gastrostomy, but we believe further invasive treatment is not necessary and prefer placing a nasogastric tube using fluoroscopy to confirm the proper positioning. 18 25 Staff must be on the alert for signs of ulceration and sentinel bleeding, while a nasogastric tube is in situ. A suspicious change in the color of gastric aspirates should be noted.

As sufficient nutritional support is essential for tissue regeneration and healing, enteral feeding should be initiated as soon as possible after the removal of LBB. Intravenous hyperalimentation is another option, but as long as the patient tolerates tube feeding, it is better to avoid complications associated with central venous nutrition. In terms of pharmacological treatment, appropriate antibiotics and antipeptic therapy must be administered during healing of the TEF. We advocate continuing antibiotics until serum inflammation markers normalize and maintaining antipeptic therapy until full oral feeding is reestablished.

Taking into account the slow healing process in spontaneous closure of TEF, a structured follow-up plan for evaluating the efficacy of the treatment is important to detect complications without delay. As a majority of cases had no evidence of TEF at the time of LBB retrieval, follow-up esophagoscopy 1 week after the procedure is essential to make an accurate diagnosis. 26 Sign of healing should be assessed at periodic follow-up endoscopy and as long as tissue healing process is observed, the conservative management can be continued up to 4 weeks. Even if the fistula appears to be closed, extra esophagoscopy is recommended to confirm the stability of healing. In case there is no evidence of tissue healing or spontaneous closure is not promising over 4 weeks, surgical intervention should be considered. A strategy of conservative management must include regular reassessment to be successful.

## Conclusion

Acquired TEF induced by LBB ingestion is still one of the most challenging issues for pediatric surgeons. Conservative management can avoid surgical intervention and related complications in selected patients. As long as the patient's systemic condition allows, we advocate conservative management as a primary treatment option and recommend it be continued for no less than 1 month, if signs of healing are observed. As hasty refeeding may be detrimental and tend to cause recurrence, esophageal rest with sufficient nutrition and careful follow-up are keys to success of conservative management.

## Notes

New Insights & the Importance for the Pediatric Surgeon This case report highlighted the successful conservative treatment of acquired tracheoesophageal fistula (TEF) due to lithium button battery (LBB) ingestion; otherwise, invasive surgery is usually performed. By reviewing previous reports, the authors summarize the unique clinical course of TEF due to LBB ingestion and suggest indications for conservative management.
